# Supplementary material for: Nucleosome landscape reflects phenotypic differences in Trypanosoma cruzi life forms
Source: PLoS Pathog. 2021 Jan 26;17(1):e1009272. doi: 10.1371/journal.ppat.1009272 (PMC7864430; doi:10.1371/journal.ppat.1009272)
Supplement: S1 Fig — A. Electropherogram of MNase digestion of 3 biological replicates from epimastigote and trypomastigote life forms. B. Scheme of the pipeline used to explore the MNase-seq data. C. Number of mapped reads against the Trypanosoma cruzi CL-Brenner Esmeraldo-like genome for each dataset (biological replicates). Blue bars represent total dataset reads; orange bars indicate the mapped reads, and gray bars show the remaining mapped reads with MAPQ scores above 10. Percentage is relative to the total reads in each dataset (blue bars). (PDF) [file ppat.1009272.s001.pdf]

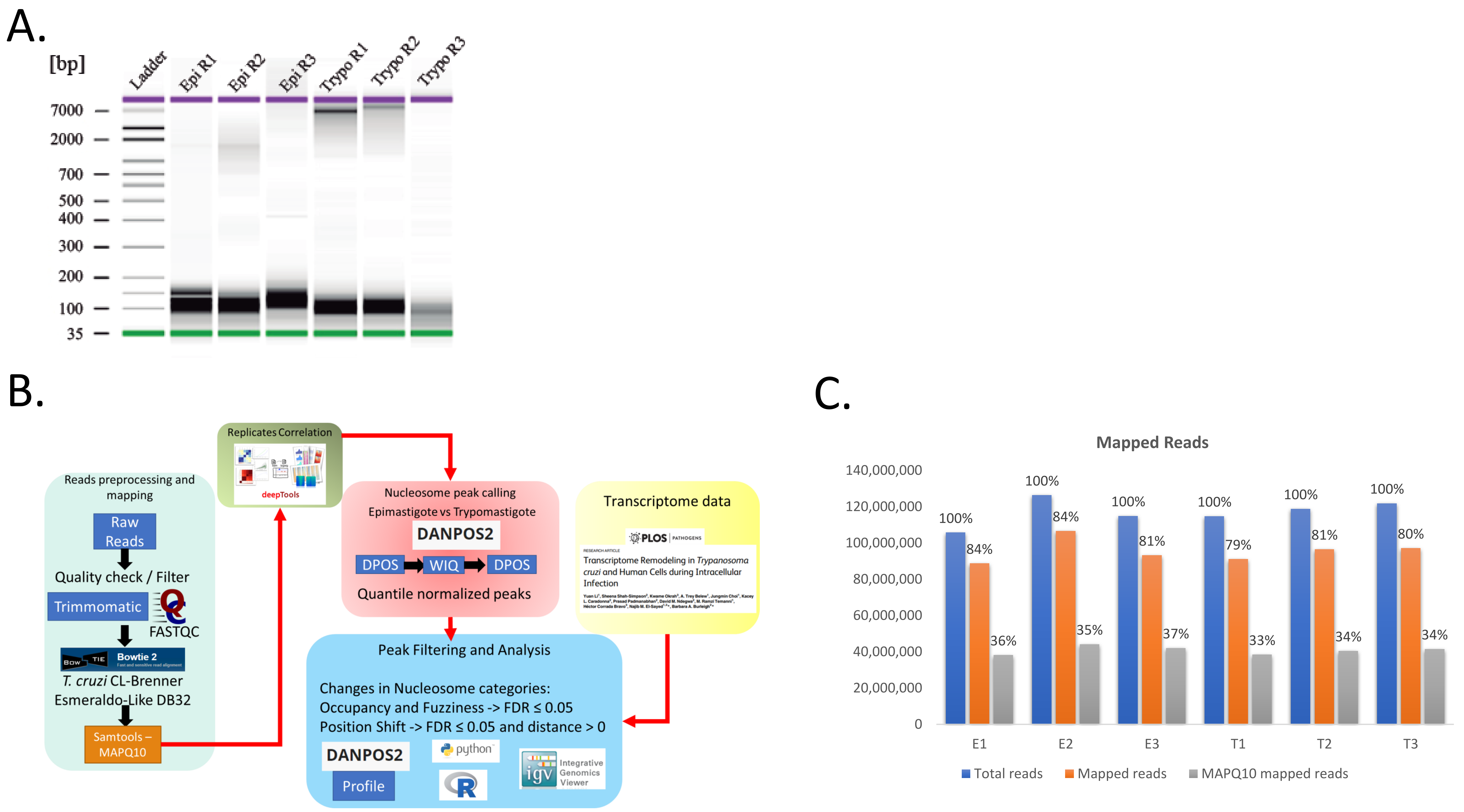

S1 Fig. A. Electropherogram of MNase digestion of 3 biological replicates from epimastigote and trypomastigote life forms. B. Scheme of the pipeline used to explore the MNase-seq data. C. Number of mapped reads against the *Trypanosoma cruzi* CL-Brenner Esmeraldo-like genome for each dataset (biological replicates). Blue bars represent total dataset reads; orange bars indicate the mapped reads, and gray bars show the remaining mapped reads with MAPQ scores above 10. Percentage is relative to the total reads in each dataset (blue bars).
