# Supplementary material for: Nucleosome landscape reflects phenotypic differences in Trypanosoma cruzi life forms
Source: PLoS Pathog. 2021 Jan 26;17(1):e1009272. doi: 10.1371/journal.ppat.1009272 (PMC7864430; doi:10.1371/journal.ppat.1009272)
Supplement: S2 Fig — A. Representative IGV snapshots of MNase-seq data mapped against the T. cruzi CL-Brener Esmeraldo-like genome (TcChr13S-386,961–415,664). Peaks represent the nucleosome occupancy level calculated by DANPOS2. Epimastigote biological replicates (R1, R2 and R3) are shown in red, and trypomastigote biological replicates (R1, R2 and R3) are shown in blue. Merged datasets of each life form are in the first two lines. The last line indicates the difference in occupancy: red peaks indicate high occupancy in epimastigotes, while blue peaks indicate higher occupancy in trypomastigotes. B. Spearman correlation of read counts for each dataset (biological replicates) from epimastigote (Epi) and trypomastigote (Trypo) life forms generated with DeepTools. (PDF) [file ppat.1009272.s002.pdf]

A.

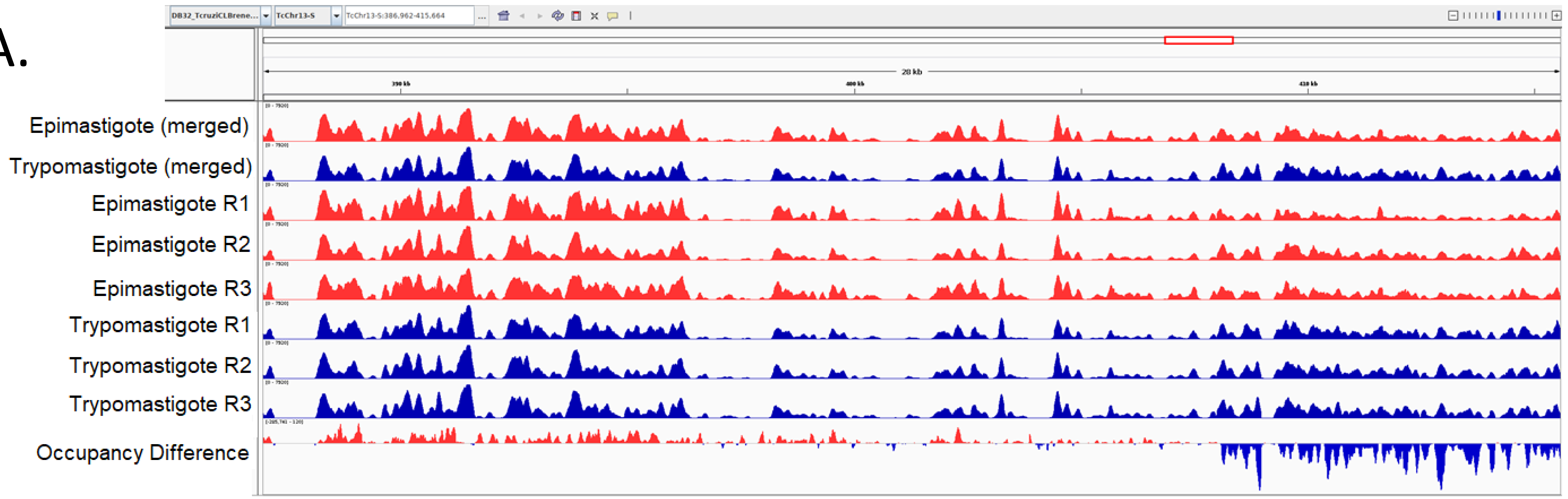

B.

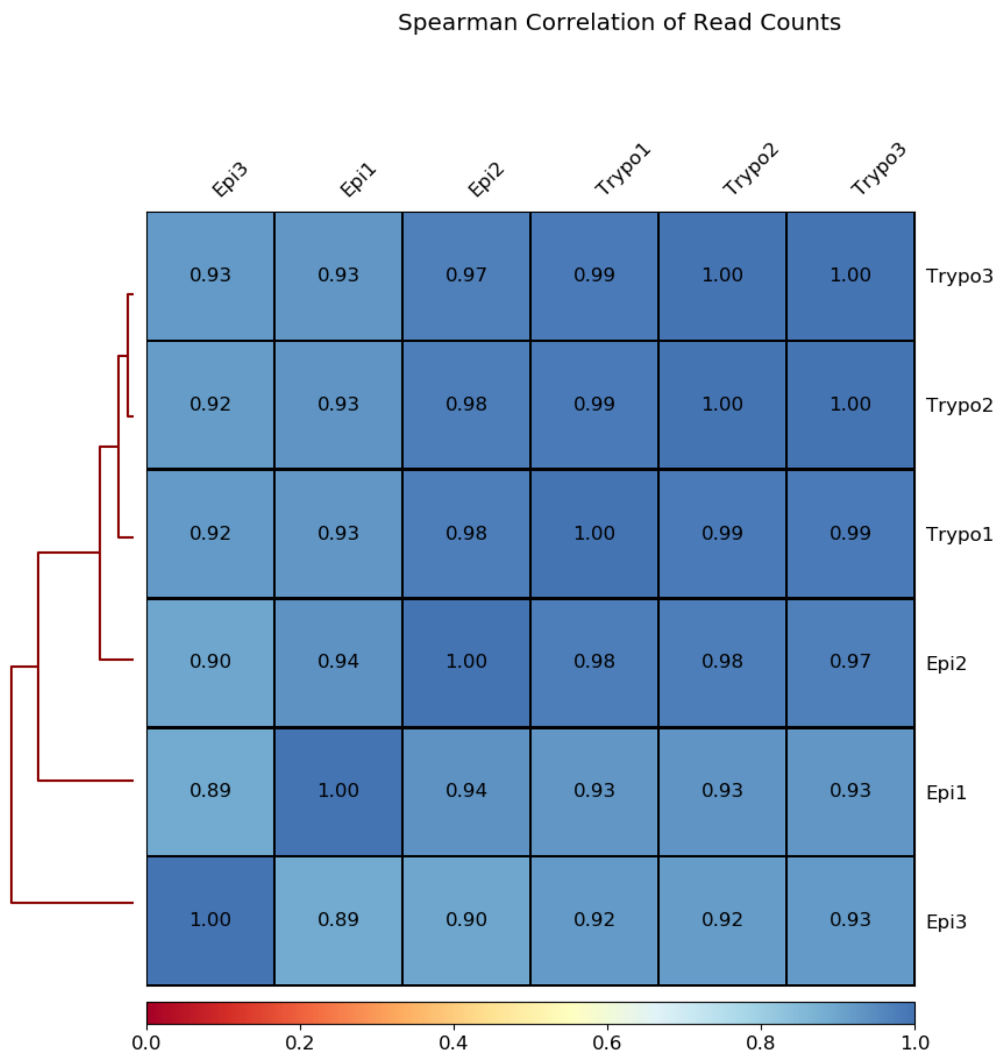

S2 Fig . A. Representative IGV snapshots of MNase-seq data mapped against the *T. cruzi* CL-Brener Esmeraldo-like genome (TcChr13S-386,961-415,664). Peaks represent the nucleosome occupancy level calculated by DANPOS2. Epimastigote biological replicates (R1, R2 and R3) are shown in red, and trypomastigote biological replicates (R1, R2 and R3) are shown in blue. Merged datasets of each life form are in the first two lines. The last line indicates the difference in occupancy: red peaks indicate high occupancy in epimastigotes, while blue peaks indicate higher occupancy in trypomastigotes. B. Spearman correlation of read counts for each dataset (biological replicates) from epimastigote (Epi) and trypomastigote (Trypo) life forms generated with DeepTools.
