# Supplementary material for: Nucleosome landscape reflects phenotypic differences in Trypanosoma cruzi life forms
Source: PLoS Pathog. 2021 Jan 26;17(1):e1009272. doi: 10.1371/journal.ppat.1009272 (PMC7864430; doi:10.1371/journal.ppat.1009272)
Supplement: S3 Fig — Only statistical residual values are represented. (PDF) [file ppat.1009272.s003.pdf]

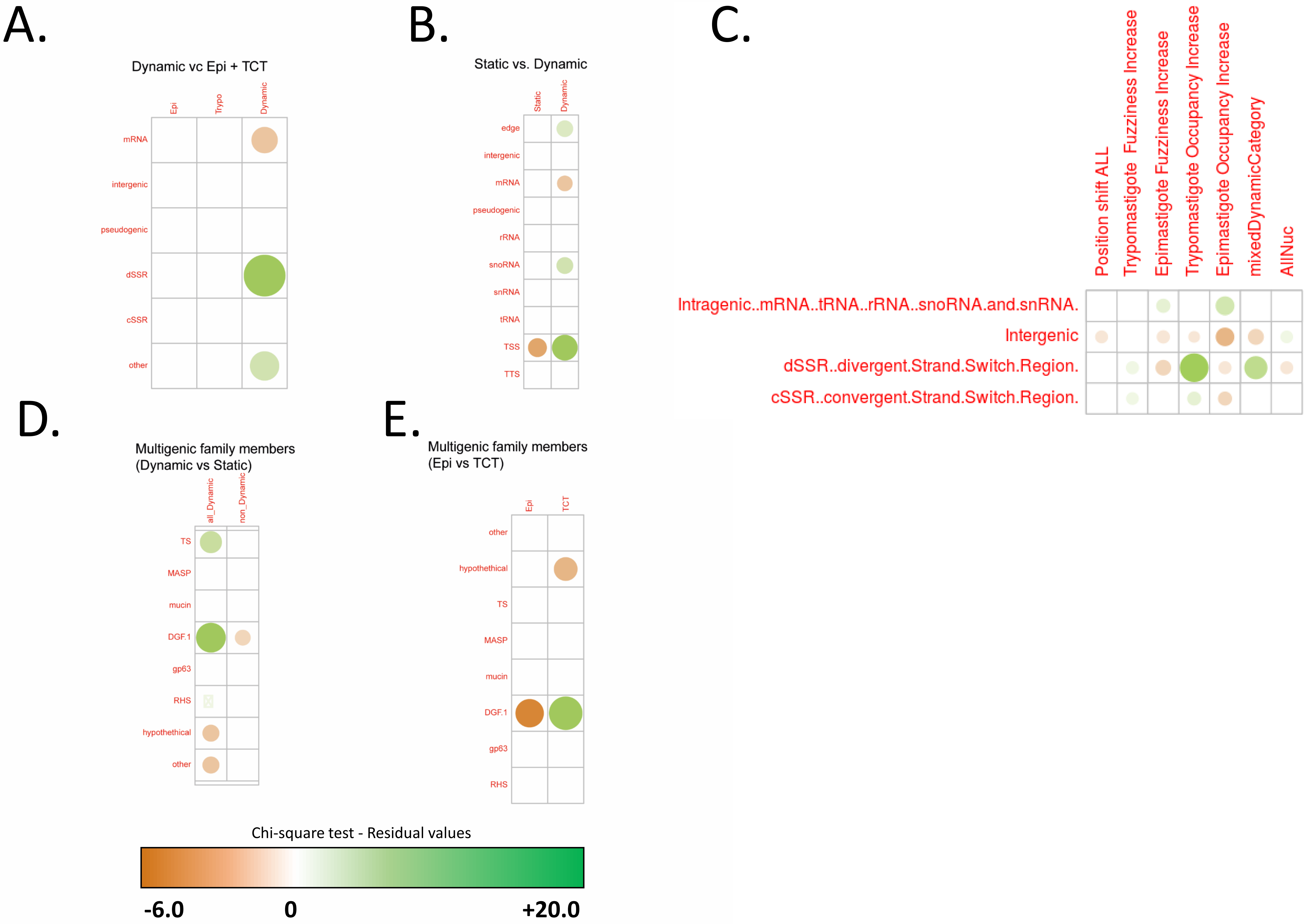

S3 Fig . Scheme of residual values from the chi-square test with Bonferroni correction (p-value <0.05) for data from Figure 1B (A), Figure 1E and S6(B), Figure S4B (C) and Figure 6B (D and E). Only statistical residual values are represented.
