# Supplementary material for: Nucleosome landscape reflects phenotypic differences in Trypanosoma cruzi life forms
Source: PLoS Pathog. 2021 Jan 26;17(1):e1009272. doi: 10.1371/journal.ppat.1009272 (PMC7864430; doi:10.1371/journal.ppat.1009272)
Supplement: S4 Fig — A. Venn diagram of the three dynamic nucleosome classes found by DANPOS2. B. Scheme of dynamic nucleosome classes obtained by DANPOS2. C. Number of dynamic nucleosomes (FDR <0.05) distributed in four genomic features for each life form according to nucleosome dynamic class. On the right, the total number in each category is shown. Here, pseudogenes were not included in intragenic regions. C. Distribution of dynamic nucleosomes classified into 3 categories in intragenic and intergenic regions. A similar distribution for dSSRs and cSSRs is shown in Fig 2B. (PDF) [file ppat.1009272.s004.pdf]

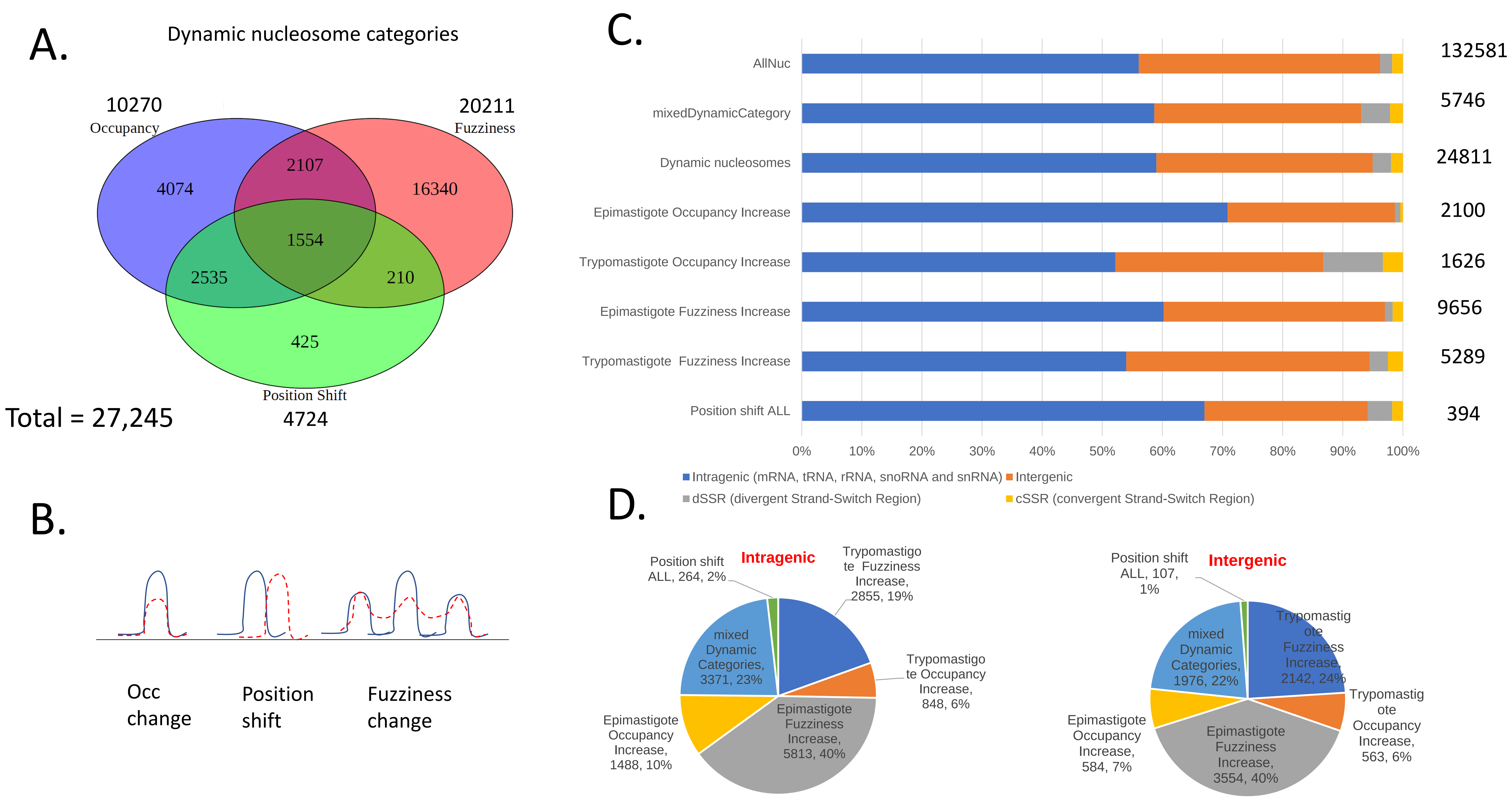

S4 Fig . Distribution of dynamic nucleosomes according to their dynamic changes. A. Venn diagram of the three dynamic nucleosome classes found by DANPOS2. B. Scheme of dynamic nucleosome classes obtained by DANPOS2. C. Number of dynamic nucleosomes (FDR <0.05) distributed in four genomic features for each life form according to nucleosome dynamic class. On the right, the total number in each category is shown. Here, pseudogenes were not included in intragenic regions. C. Distribution of dynamic nucleosomes classified into 3 categories in intragenic and intergenic regions. A similar distribution for dSSRs and cSSRs is shown in Fig 2B.
