# Supplementary material for: Nucleosome landscape reflects phenotypic differences in Trypanosoma cruzi life forms
Source: PLoS Pathog. 2021 Jan 26;17(1):e1009272. doi: 10.1371/journal.ppat.1009272 (PMC7864430; doi:10.1371/journal.ppat.1009272)
Supplement: S5 Fig — Changes in fuzziness (A), occupancy levels (B) and position shift (C) are depicted. Distances are based from -200 bp to +200 bp of the dyad (represented by 0 on the x axis). Trypomastigotes are represented in blue, and epimastigotes are represented in red. D. Histogram showing the frequency of position shifts detected between life forms. (PDF) [file ppat.1009272.s005.pdf]

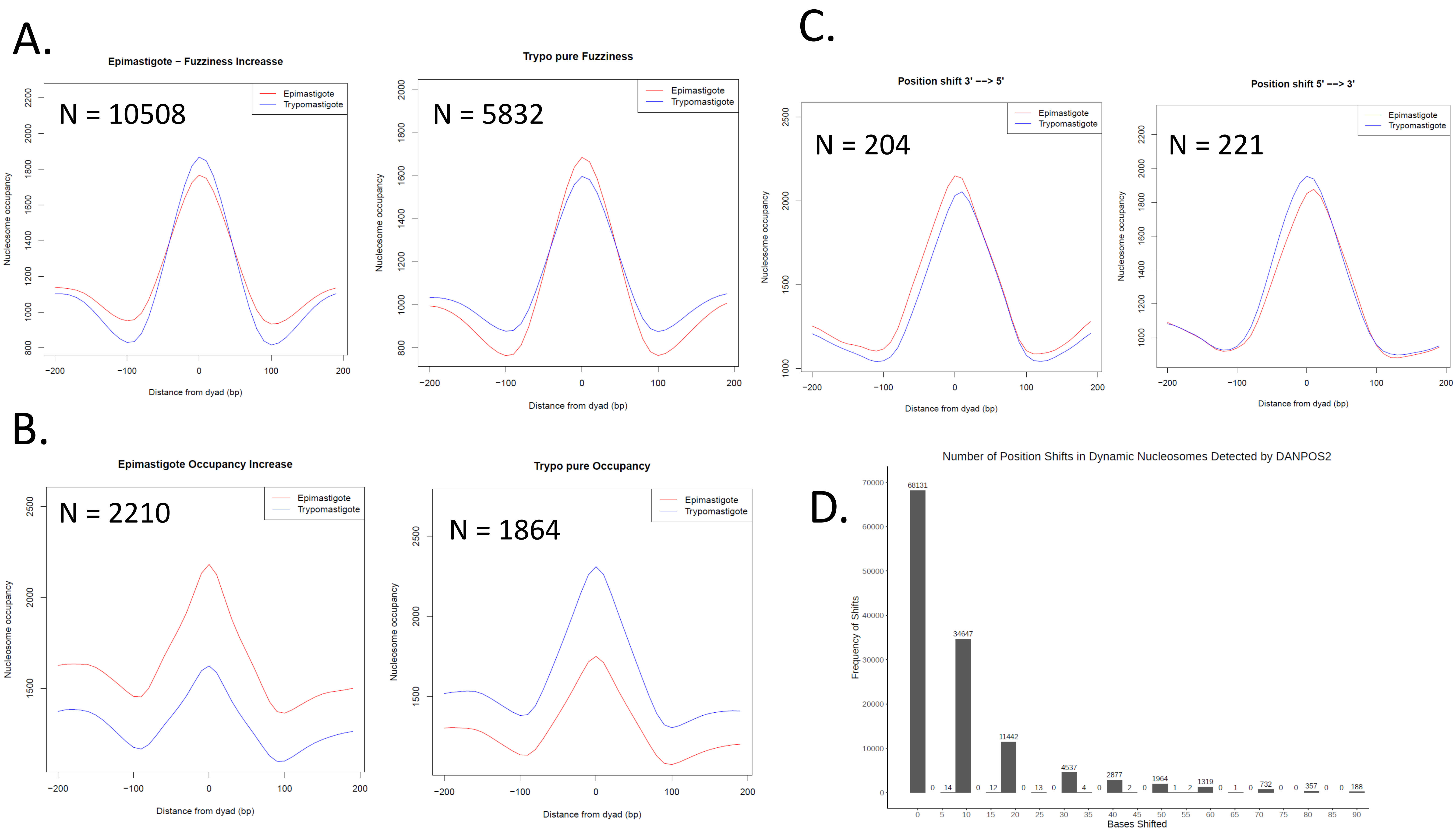

S5 Fig. Occupancy plots for each dynamic nucleosome class are presented in Figure S4B. Changes in fuzziness (A), occupancy levels (B) and position shift (C) are depicted. Distances are based from -200 bp to +200 bp of the dyad (represented by 0 on the x axis). Trypomastigotes are represented in blue, and epimastigotes are represented in red. D. Histogram showing the frequency of position shifts detected between life forms.
