# Supplementary material for: Nucleosome landscape reflects phenotypic differences in Trypanosoma cruzi life forms
Source: PLoS Pathog. 2021 Jan 26;17(1):e1009272. doi: 10.1371/journal.ppat.1009272 (PMC7864430; doi:10.1371/journal.ppat.1009272)
Supplement: S6 Fig — A. Percentage of dynamic and static nucleosomes (sum of nucleosomes from both life forms) at the indicated features. * significant differences using chi-square test under α = 0.05 with corrected z-score = +/- 2.8 by Bonferroni; with residual +3.7 for snoRNA (overrepresented at dynamic category). B. The diff wig file obtained from DANPOS2 analysis was used to extract nucleosome occupancy difference between Epimastigote and TCT. The dSSR bed file produced in this work was used, along with the pyBigWig library, to extract the occupancy difference values for each dSSR region and to calculate their average value, which are plotted in this histogram. C. TPM counts from epimastigotes and TCTs polycistrons were obtained from Li et al (2016). The top 10% dSSR with highest difference in nucleosome occupancy (obtained from B) is highlight in red (epimastigote>TCT) and blue (TCT>epimastigote). (PDF) [file ppat.1009272.s006.pdf]

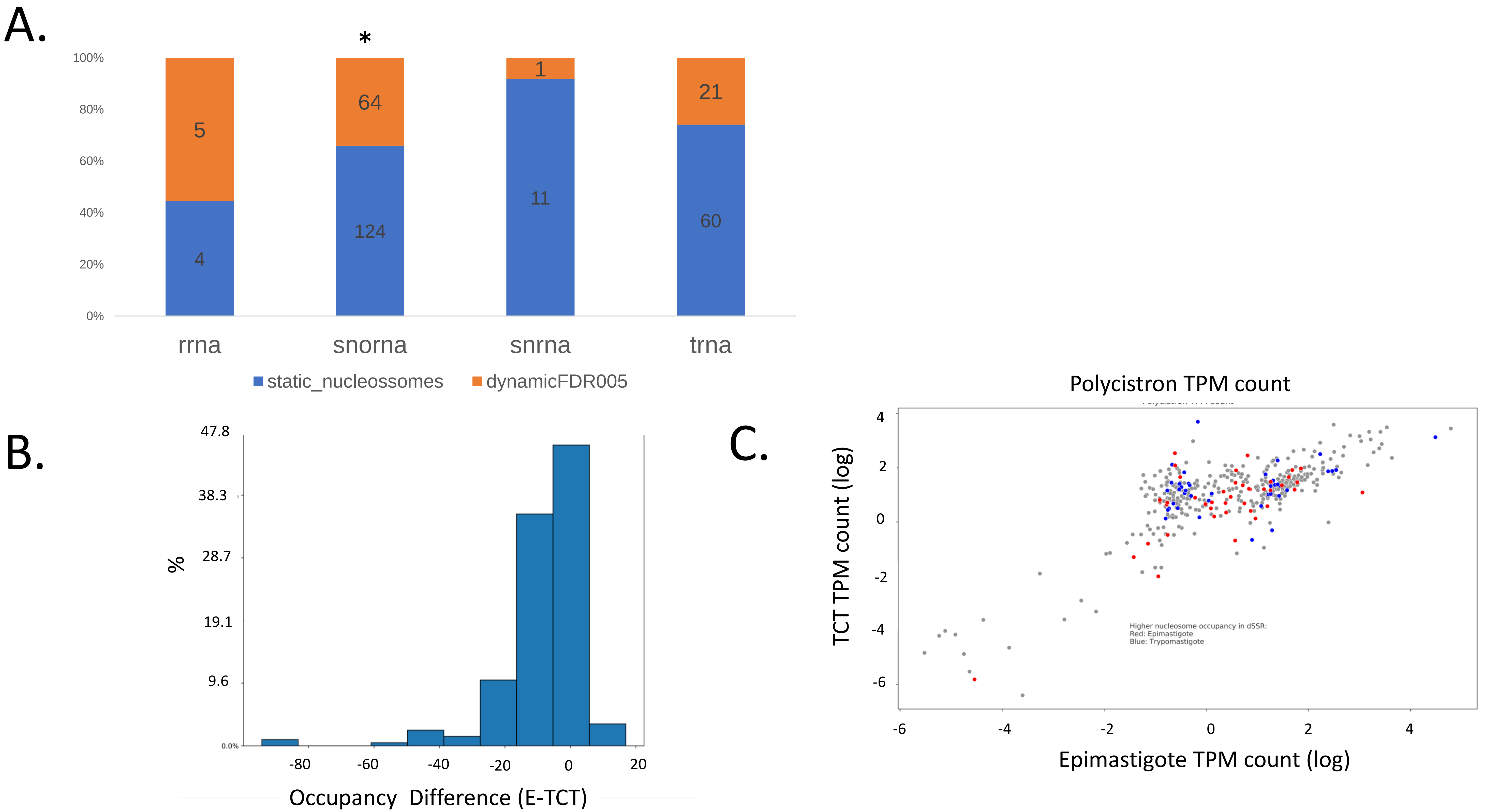

S6 Fig. A. Percentage of dynamic and static nucleosomes (sum of nucleosomes from both life forms) at the indicated features. * significant differences using chi-square test under α=0.05 with corrected z-score = +/- 2.8 by Bonferroni; with residual +3.7 for snoRNA (overrepresented at dynamic category). **B.** The diff wig file obtained from DANPOS2 analysis was used to extract nucleosome occupancy difference between Epimastigote and TCT. The dSSR bed file produced in this work was used, along with the pyBigWig library, to extract the occupancy difference values for each dSSR region and to calculate their average value, which are plotted in this histogram. **C.** TPM counts from epimastigotes and TCTs polycistrons were obtained from Li et al (2016). The top 10% dSSR with highest difference in nucleosome occupancy (obtained from B) is highlight in red (epimastigote>TCT) and blue (TCT>epimastigote).
