# Supplementary material for: Nucleosome landscape reflects phenotypic differences in Trypanosoma cruzi life forms
Source: PLoS Pathog. 2021 Jan 26;17(1):e1009272. doi: 10.1371/journal.ppat.1009272 (PMC7864430; doi:10.1371/journal.ppat.1009272)
Supplement: S7 Fig — Here, dynamic nucleosomes were obtained from 1000 bp upstream and 500 bp downstream from the first ATG of the CDS of the first polycistron. (PDF) [file ppat.1009272.s007.pdf]

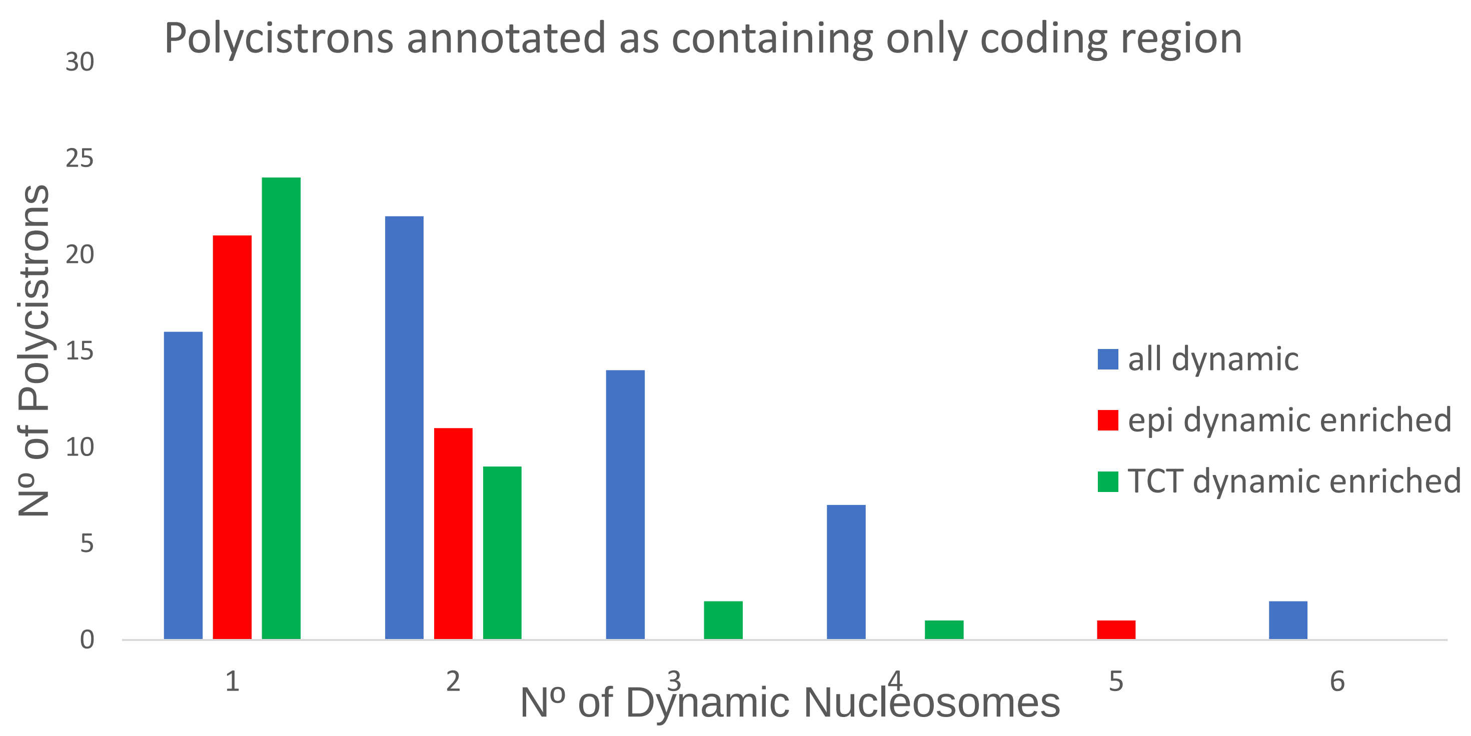

S7 Fig . Distribution of polycistrons coding for only one gene according to the number of dynamic nucleosomes found at their associated TSS. Here, dynamic nucleosomes were obtained from 1000 bp upstream and 500 bp downstream from the first ATG of the CDS of the first polycistron.
