# Supplementary material for: Nucleosome landscape reflects phenotypic differences in Trypanosoma cruzi life forms
Source: PLoS Pathog. 2021 Jan 26;17(1):e1009272. doi: 10.1371/journal.ppat.1009272 (PMC7864430; doi:10.1371/journal.ppat.1009272)
Supplement: S8 Fig — A. GO terms (biological process) enriched (p<0.05) for IDs with static (FDR< 0.01) nucleosomes (6980) and for IDs with dynamic nucleosomes (3499). For static IDs, all GO terms are shown, while for dynamic IDs, only the top 20 terms are shown (filtered by those whose GO terms harbor fewer than 8 members). Full GO terms, p-values, fold enrichment and IDs are shown in the S5 Table. B. The top 100 dynamic IDs (ranked by number of dynamic nucleosomes per kbp) were searched for GO/REVIGO analysis annotation using TriTrypDB tools. The scatterplot shows a clusterization of GO terms (remaining after a redundancy reduction) in a two-dimensional semantic space, resulting in similar terms being plotted next to each other according to [75]. In general, more semantically similar GO terms are closer in the plot. C. Venn diagram of IDs with increased nucleosome dynamics (occupancy and/or fuzziness) in epimastigotes and TCTs. (PDF) [file ppat.1009272.s008.pdf]

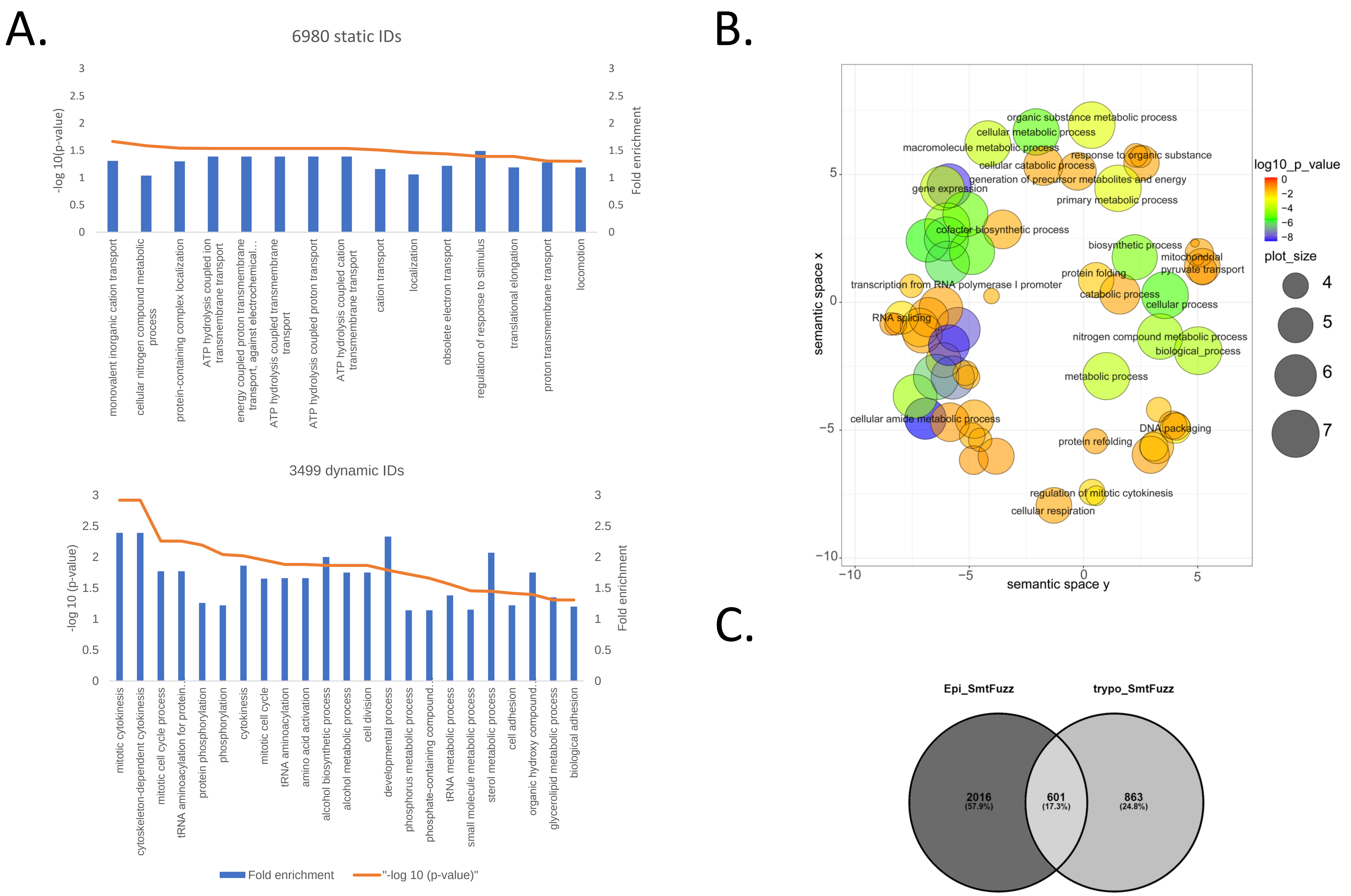

S8 Fig. A. GO terms (biological process) enriched (p<0.05) for IDs with static (FDR< 0.01) nucleosomes (6980) and for IDs with dynamic nucleosomes (3499). For static IDs, all GO terms are shown, while for dynamic IDs, only the top 20 terms are shown (filtered by those whose GO terms harbor f than 8 members). Full GO terms, p-values, fold enrichment and IDs are shown in the Supplemental Table. **B.** The top 100 dynamic IDs (ranked by number of dynamic nucleosomes per kbp) were searched for GO/REVIGO analysis annotation using TriTrypDB tools. **C.** Venn diagram of IDs with increased nucleosome dynamics (occupancy and/or fuzziness) in epimastigotes and TCTs.
