# Supplementary material for: Nucleosome landscape reflects phenotypic differences in Trypanosoma cruzi life forms
Source: PLoS Pathog. 2021 Jan 26;17(1):e1009272. doi: 10.1371/journal.ppat.1009272 (PMC7864430; doi:10.1371/journal.ppat.1009272)
Supplement: S9 Fig — (PDF) [file ppat.1009272.s009.pdf]

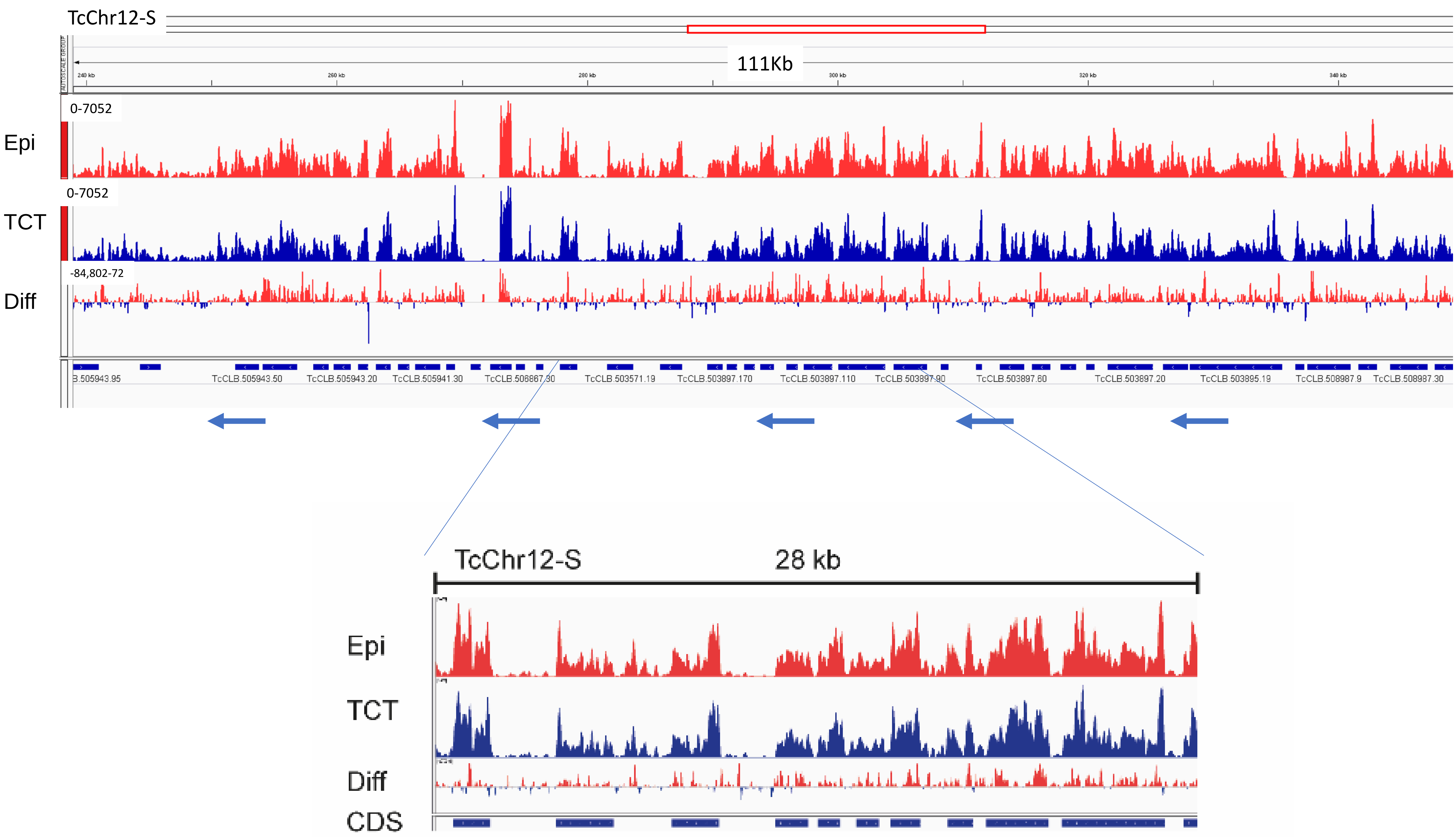

S9 Fig. Representative IGV snapshots of MNase-seq data mapped against the *T. cruzi* CL-Brener Esmeraldo-like genome at Chr 12S highlighting a CDS-containing region. Arrows indicate transcription direction.
