# Supplementary material for: Nucleosome landscape reflects phenotypic differences in Trypanosoma cruzi life forms
Source: PLoS Pathog. 2021 Jan 26;17(1):e1009272. doi: 10.1371/journal.ppat.1009272 (PMC7864430; doi:10.1371/journal.ppat.1009272)
Supplement: S11 Fig — A. Total length, in kbp, of members of the multigenic family. B. Number of dynamic and total (dynamic plus static) nucleosomes per kbp in multigenic family members, hypothetical genes and others (refers to all other IDs, including tRNA, rRNA, snRNA, snoRNA, mRNA, and pseudogene). C. Distribution of all nucleosomes, all dynamic nucleosomes, and dynamic nucleosomes in TCTs and epimastigotes (increase at occupancy and/or fuzziness) with regard to the distribution in disrupted and conserved T. cruzi genome compartments. DGF-1, GP63 and RHS are expected to be found in both compartments according to Berná et al. (2008). D. Nucleosomes per kbp. Conserved compartments have slightly more nucleosomes per kbp. (PDF) [file ppat.1009272.s011.pdf]

A.

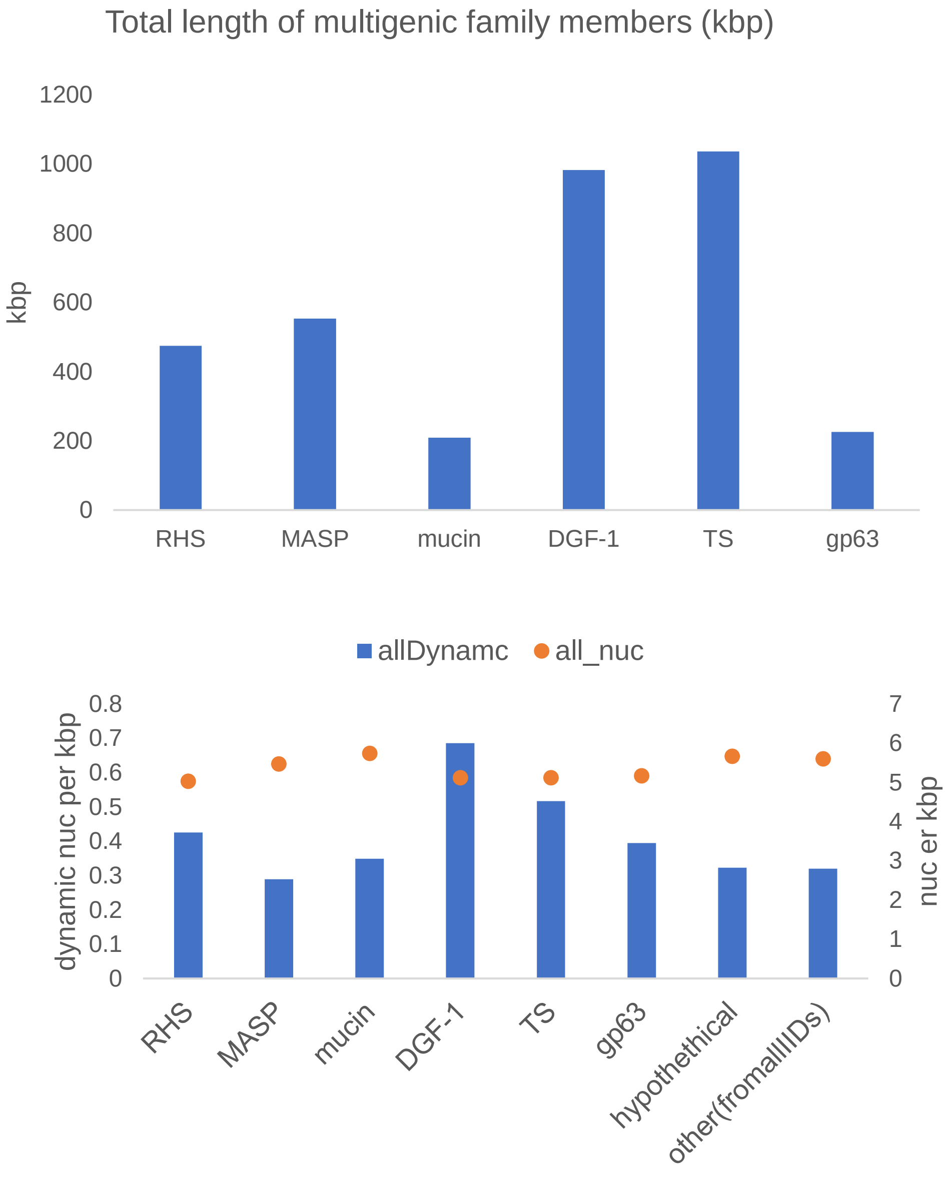

B.

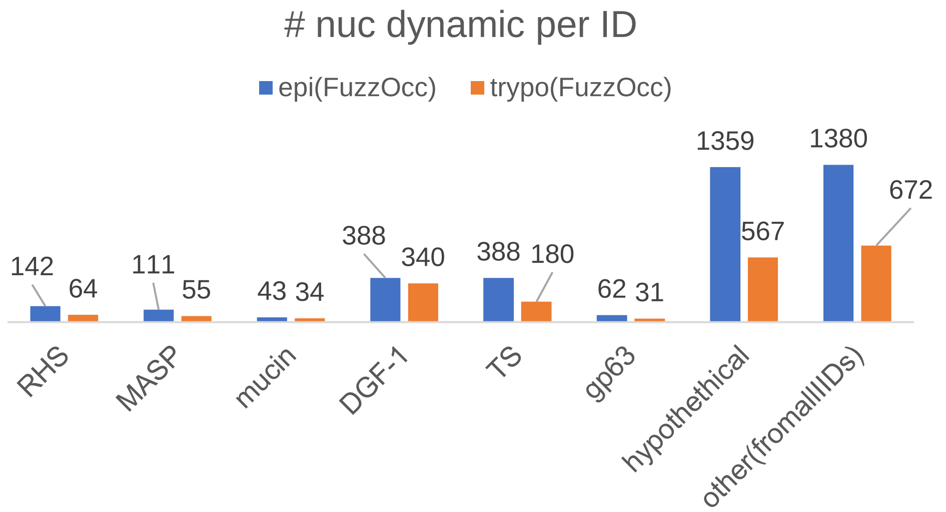

C.

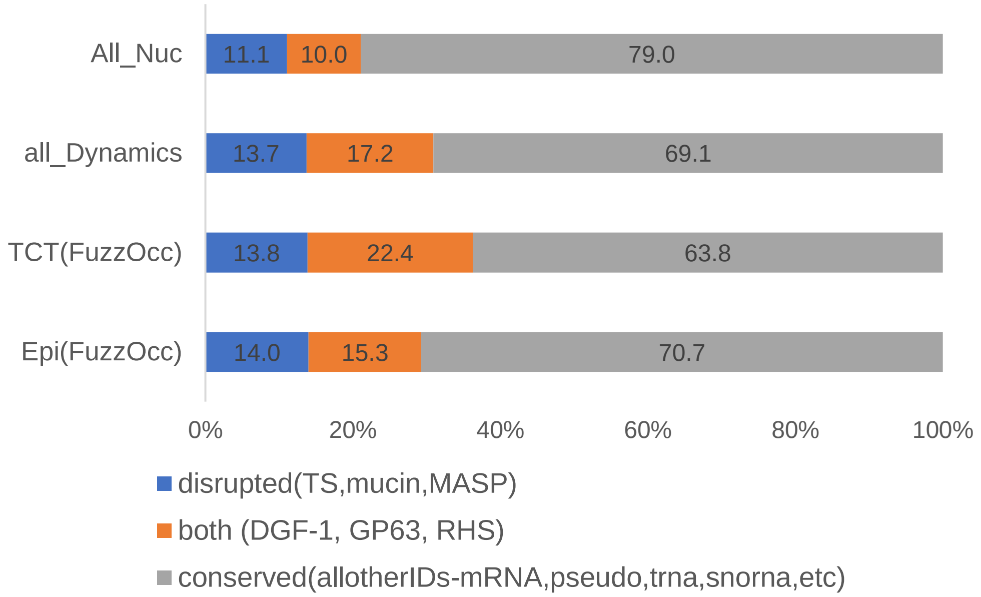

D.

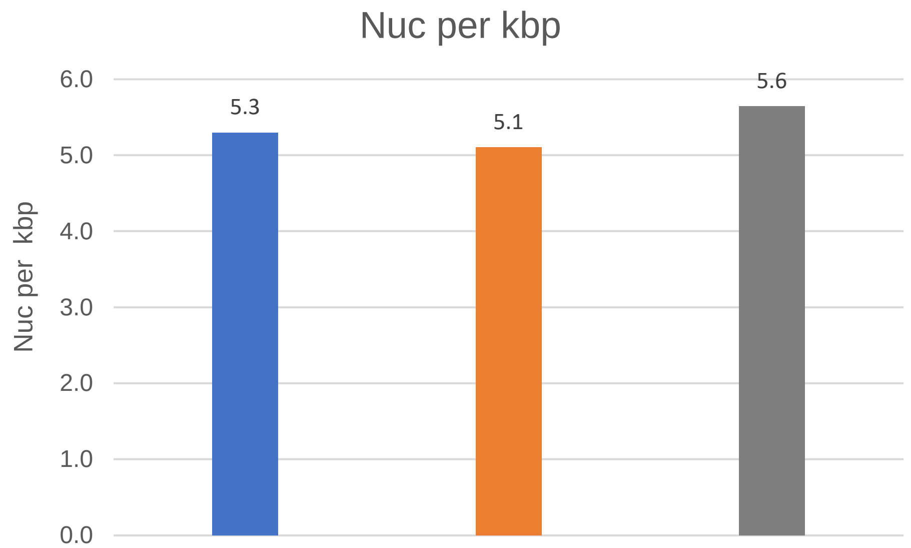

S11 Fig. A. Total length, in kbp, of members of the multigenic family (top). Bottom, number of dynamic and total (dynamic plus static) nucleosomes per kbp in multigenic family members, hypothetical genes and others (refers to all other IDs, including tRNA, rRNA, snRNA, snoRNA, mRNA, and pseudogene). **B.** Number of dynamic nucleosomes per ID at the same categories described above. **C.** Distribution of all nucleosomes, all dynamic nucleosomes, and dynamic nucleosomes in TCTs and epimastigotes (increase at occupancy and/or fuzziness) with regard to the distribution in disrupted and conserved *T. cruzi* genome compartments. DGF-1, GP63 and RHS are expected to be found in both compartments according to [1]. **D.** Nucleosomes per kbp. Conserved compartments have slightly more nucleosomes per kbp.

Reference

[1] Berná L, Rodriguez M, Chiribao ML, Parodi-Talice A, Pita S, Rijo G, et al. Expanding an expanded genome: long-read sequencing of Trypanosoma cruzi. Microbial genomics. 2018;4(5). Epub 2018/05/01. doi: 10.1099/mgen.0.000177. PubMed PMID: 29708484; PubMed Central PMCID: PMCPMC5994713
