# Supplementary material for: Nucleosome landscape reflects phenotypic differences in Trypanosoma cruzi life forms
Source: PLoS Pathog. 2021 Jan 26;17(1):e1009272. doi: 10.1371/journal.ppat.1009272 (PMC7864430; doi:10.1371/journal.ppat.1009272)
Supplement: S10 Fig — A. Density of nucleosome occupancy (values on the y axis from Figs 4A and 5A–5H) for the indicated gene classes/features in epimastigotes (pink) and TCTs (green). The overlap percentage between life forms is indicated above each density plot. B. Box-plot of point by point distance (in y axis) from epimastigote and TCT landscapes (from Figs 4A and 5A–5H). A Tukey test was performed (considering intergenic regions as a control with few changes between epimastigotes and TCTs) to verify which gene classes/features have more differences between life forms. C. Representation of the results (95% confidence interval) of a pairwise Tukey test (intergenic as a control). Comparisons close to zero are considered not significantly different. (PDF) [file ppat.1009272.s010.pdf]

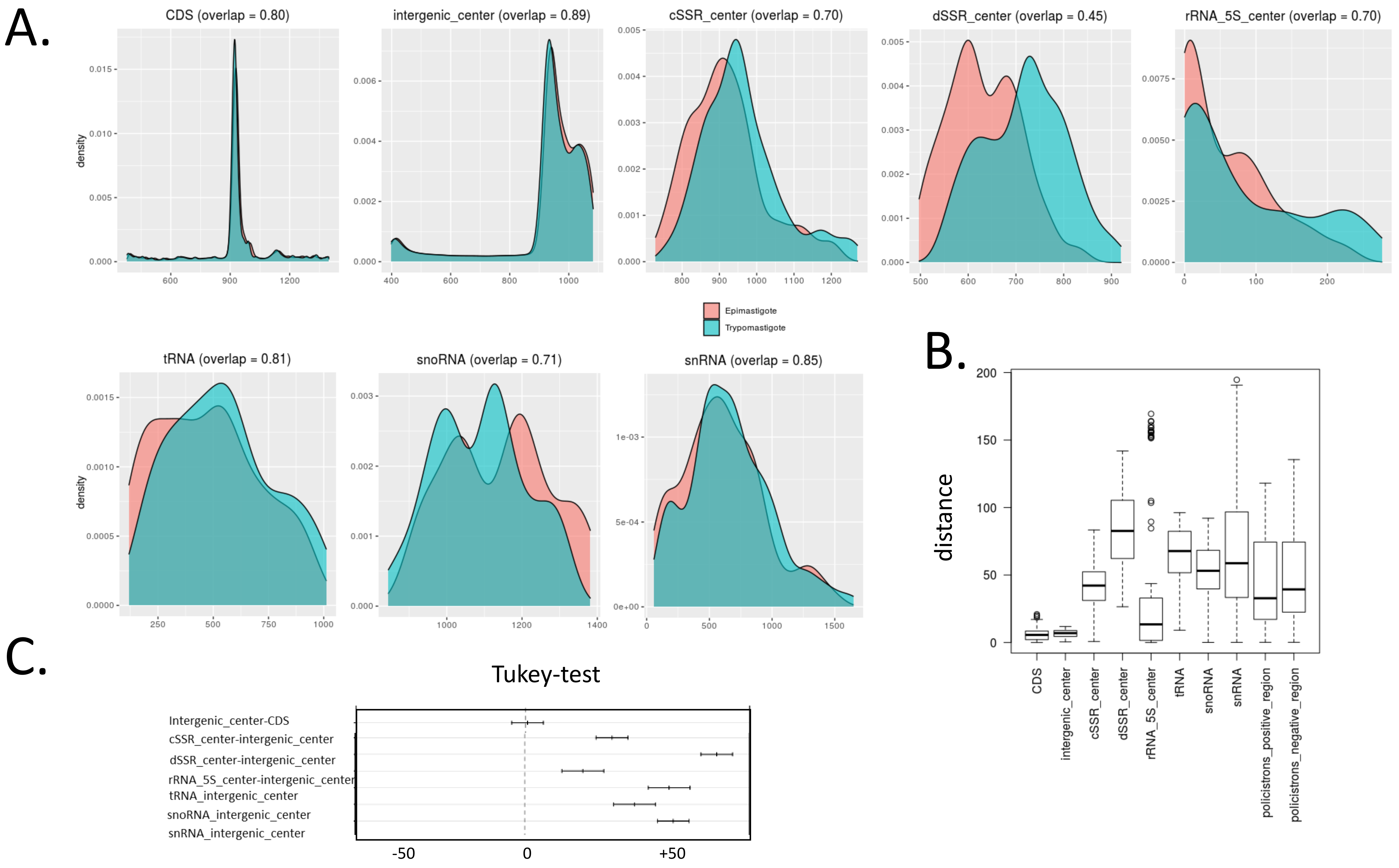

S10 Fig. A. Density of nucleosome occupancy (values on the y axis from Figure 4A, 5A-H) for the indicated gene classes/features in epimastigotes (pink) and TCTs (green). The overlap percentage between life forms is indicated above each density plot. **B**. Box-plot of point by point distance (in y axis) from epimastigote and TCT landscapes (from Figure 4A, 5A-H). A Tukey test was performed (considering intergenic regions as a control with few changes between epimastigotes and TCTs) to verify which gene classes/features have more differences between life forms. **C**. Representation of the results (95% confidence interval) of a pairwise Tukey test (intergenic as a control). Comparisons close to zero are considered not significantly different.
