# Supplementary material for: Nucleosome landscape reflects phenotypic differences in Trypanosoma cruzi life forms
Source: PLoS Pathog. 2021 Jan 26;17(1):e1009272. doi: 10.1371/journal.ppat.1009272 (PMC7864430; doi:10.1371/journal.ppat.1009272)
Supplement: Attachment — Submitted filename: AnswersFinalok.doc [file ppat.1009272.s018.doc]

**São Paulo, 12/15/2020**

**Dear editor,**

**Dr. Christine Clayton**

We thank the editors and the referees for carefully read our manuscript and making important questions and suggestions. We have answered all questions accordingly, and we are confident that the manuscript is now more robust and clearer. We hope that this manuscript meets the high standards of PlosPathogens.

This new version includes two new figures: Figure S6 B-C (B- a histogram of differential (E-TCT) nucleosome occupancy in all dSSRs; C- TPM values across all polycistronic regions) and Figure S9 (a zoom out and zoom in from previous Figure 5H top). These analyses were suggested by the revisor 2 and 1, respectively. In addition, we corrected some typos all over the manuscript, which can be seen at the tracked changed document version.

We also add Dr. Ariel Mariano Silber as an author for this manuscript. Dr. Silber contributed constantly for this project conceptualization and development. His contribution was further evidenced upon this first round of revision. Therefore, we decided to include him as an author.

            Please do not hesitate to contact us if you need any further information.

**Please find below, the answers for all referee’s questions.**

Sincerely,

M.Carolina Elias, PhD and Julia Cunha, PhD

Av. Vital Brasil, 1500. CEP: 05338-000

São Paulo-SP

+55-11-26279731

Dear Dr da Cunha,

Thank you very much for submitting your manuscript "Nucleosome landscape reflects phenotypic differences in Trypanosoma cruzi life forms" for consideration at PLOS Pathogens. As with all papers reviewed by the journal, your manuscript was reviewed by members of the editorial board and by several independent reviewers. The reviewers appreciated the attention to an important topic. Based on the reviews, we are likely to accept this manuscript for publication, providing that you modify the manuscript according to the review recommendations.

The detailed comments are appended and most are very useful.

One short point from me (Christine Clayton): transcriptional down-regulation in non-dividing forms of kinetoplastids is not unprecedented. Trypanosoma brucei stumpy forms have almost no incorporation of 3H uridine into mRNA and also have very low levels of mRNA (E. Pays et al., Abrupt RNA changes precede the first cell division during the differentiation of Trypanosoma brucei bloodstream forms into procyclic forms in vitro. Mol. Biochem. Parasitol. 61, 107-114 (1993)). It is also impossible to induce expression from an RNA pol I promoter in the stumpy form (see e.g. B. Liu, K. Marucha, C. Clayton, The zinc finger proteins ZC3H20 and ZC3H21 stabilise mRNAs encoding membrane proteins and mitochondrial proteins in insect-form Trypanosoma brucei. Molecular microbiology 113, 430-451 (2020). Transcriptome results also suggest low activity in metacyclic forms, though no measurements of transcription have been made. I think you should refer at least to the Pays paper, since their results are consistent with your hypothesis for T. cruzi non-dividing forms.

# *We appreciate your comments and suggestions. In addition to the suggested papers to be cited, we found that global levels of protein synthesis and mRNA levels were previously measured at metacyclics- also a non-dividing cell, and compared to procyclics (“The proteome and transcriptome of the infectious metacyclic form of Trypanosoma brucei define quiescent cells primed for mammalian invasion” -* [***Christiano***](https://pubmed.ncbi.nlm.nih.gov/?sort=date&term=Christiano+R&cauthor_id=28742275) ***R et al; 2017).***

# ***Therefore, we added the following sentence at Introduction:***

*“Likewise, in T. brucei stumpy and metacyclic (both non-replicative) forms have almost no incorporation of 3H uridine into nascent mRNAs and 35S cysteine incorporation into newly translated protein, respectively, and both have very low levels of mRNAs [1, 2]. In addition, it is also impossible to induce expression from an RNA pol I promoter in the stumpy form[3].”*

Reviewer Comments (if any, and for reference):

Reviewer's Responses to Questions

Part I - Summary

Please use this section to discuss strengths/weaknesses of study, novelty/significance, general execution and scholarship.

Reviewer #1: The authors present genome-wide nucleosome positioning maps in two life forms of Trypanosoma cruzi that display markedly different cell morphology, transcription and replication activity, and infectivity. Trypanosomes are an ancient eukaryotic branch that exhibit interesting genomic and epigenetic peculiarities, such as constitutive transcription of genes by RNA Pol II from long polycistronic gene clusters and an apparent absence of transcriptional regulation at the level of initiation. Similar genome-wide nucleosomal studies were published in related kinetoplastids L. major (Lombraña et al. 2016) and T. brucei (Maree et al. 2017, Wedel et al. 2017). Although some of the findings described here have been previously reported in these related organisms, the authors present novel data showing that gene classes associated with specific life forms are differentially enriched with dynamic nucleosomes. Integration of transcriptomic data revealed that these differential nucleosomal occupancies and positioning may likely be associated with important phenotypic differences in this organism. Interestingly, the authors show a correlation between nucleosome dynamics of specific gene classes and dSSRs, and RNA transcript levels and postulates that differential chromatin structures at these sites may influence RNA transcript levels.

Reviewer #2: Overall, this is a valuable study, which adds much-needed understanding to how gene expression is influenced by the organisation of the T. cruzi genome. The authors perform sequencing of MNase-digested DNA from replicating and non-replicating T. cruzi life cycle forms to evaluate nucleosome patterns across the parasite’s genome. Though this mapping approach has been described in the related trypanosomatids Leishmania and T. brucei, these data are valuable for two main reasons: (i) the T. cruzi genome is the least well understood of these trypanosomatids, due to the very large numbers of widely dispersed multigene families; and (ii) the paper asks, for the first time in trypanosomatids, if nucleosome organisation is altered in non-replicating cells, perhaps reflecting changes in gene expression. Despite this broad interest and importance, aspects of the paper could be improved.

Reviewer #3: This is a very well conducted and in-depth study that contributes to understanding the epigenetic regulation that accompanies life stage changes in Trypanosoma cruzi. The authors performed a genome-wide high-resolution nucleosome mapping comparing epimastigotes and trypomastigotes. Given the unique gene expression regulation mechanism in these parasites, mainly centered at the post-transcriptional level, the presented data is particularly significant. The researchers provided a thorough analysis of the nucleosomes landscape in both life stages and examined relevant biological issues. As stated by the authors, "results indicate that chromatin architecture, defined primarily by nucleosome positioning and occupancy, reflects the phenotypic differences found among T. cruzi life forms despite the lack of a canonical transcriptional control context." Therefore, this manuscript brings novel and relevant results and worthy of publication in PLoS Pathogens.

Part II – Major Issues: Key Experiments Required for Acceptance

Please use this section to detail the key new experiments or modifications of existing experiments that should be absolutely required to validate study conclusions.

Generally, there should be no more than 3 such required experiments or major modifications for a "Major Revision" recommendation. If more than 3 experiments are necessary to validate the study conclusions, then you are encouraged to recommend "Reject".

Reviewer #1: None

Reviewer #2: The authors define (lines 100-102) the ‘strand switch regions’ (SSRs) that surround PTUs are being sites where transcription either converges (terminates, TTSs) or diverges (initiates, TSSs), and use this definition to locate SSRs in the genome based on CDS orientation (lines 177-179). Mapping of RNA polymerase II and histone variants in Leishmania and T. brucei tells us that there is another form of SSR, which is located within a directional gene cluster and where transcription of one PTU initiates and another PTU terminates (ie adjacent TSS and TTS sites). The authors’ approach ignores these SSRs, if they exist in T. cruzi. Two things need to be considered in this light. 1. Are there no histone variant ChIP data for T. cruzi that would allow these intra-directional gene cluster SSRs to be defined? If so, all data should be re-evaluated. 2. If such data is not available, the authors must explain this limitation in their approach (first results section, materials and methods), and should discuss in what ways the absence of being able to identify such SSRs might impact on the downstream data they present.

*There is no published T. cruzi ChIP-seq data of histone variant and RNA Pol II. Recently, we have obtained H2BV ChIP-seq data, but we are currently working on that. Data visual inspection indicates that H2B variant demarcates T. cruzi TSSs as observed on T. brucei. We can observe enrichment inside polycistrons, which could suggest TSSs at non-SSRs. As this is a preliminary data analysis, we are not fully confident to considered them as putative TTSs at non-SSRs. Thus, we agree with the reviewer that our computational strategy does not considered this TSS category. Therefore, we wrote the following sentence at the Results part:*

*“ Genome mapping of histone variants (H2A.Z) and RNA Pol II enrichment as well as transcriptomics analysis of small primary transcripts carrying a 5’triphosphate and nuclear run-on assays indicate the existence of TSSs at a non-dSSR [4-6]. Due to the lack of similar datasets in T. cruzi, our strategy was focused only on the TSS found at dSSR.”.*

*We also wrote the following sentence at Discussion part:*

*“As highlighted previously, our computational strategy considered only TSSs located within dSSRs. Thus, it remains to be elucidated whether TSSs located at non-dSSRs also harbor more dynamic nucleosomes as soon as they were identified in T. cruzi.* “

Through a number of pieces of analysis, the authors note that there is greater nucleosome occupancy at divergent SSRs in trypomastigotes compared with epimastogotes, and they suggest that this may be due to decreased transcription in the former rather than latter (e.g. ‘possibly reflecting less elongation of RNA Pol II through adjacent PTUs’, line 239). If this was true, it would represent control at the point of transcription initiation, affecting a whole PTU, which would be truly very interesting (indeed, an ‘unprecedented’ observation, line 248). However, is it true? The authors have used RNA derived from the two life cycle stages for other analysis, so they should map these data and ask if they observe changes in transcript levels across whole PTUs in the two life cycle, which would be predicted from their observation. If such an effect is seen, their arguments are strengthened; if not, they should explain why not and suggest alternatives for the nucleosome patterns.

*First, we have to considered that this question would be more faithfully answered by global nascent RNA sequencing analysis (GRO-seq) [7] once transcriptomic assays reflects the steady state transcript levels after many posttranscriptional regulation, which include stabilization and degradation. GRO-seq datasets are not available for T. cruzi. To evaluate the revisor question, we have selected the* *top 10% dSSR with highest difference in nucleosome occupancy between life forms by using the “diff wig” output from DANPOS2. Polycistrons annotated adjacent to these selected dSSRs were recovered to check their TPM (transcripts per kilobase per million) count based on the LI transcriptomic study [8]. The TPM values (in log scale) for each polycistron (in both lifeforms) were plotted and those associated to the top 10% dSSR with highest difference in nucleosome were highlighted. No clear correlation between a higher occupancy in dSSR and lower TPM counts (and vice-versa) was observed.*

*We have included this analysis on the new version of the manuscript (Figure S6B and C). We have also added the following paragraph at the results section:*

*“The top 10% dSSR with the highest difference in nucleosome occupancy between life forms (S6B Fig) were compared to the TPM (transcripts per kilobase per million) counts in epimastigotes and TCTs of their flanking polycistrons - obtained from a public transcriptomic study [8]. No clear correlation between higher occupancy in dSSR and lower TPM counts (and vice-versa) was observed (S6C Fig). However, to faithfully evaluate the effect of dSSR nucleosome occupancy on RNA Pol II elongation, a global RNA nascent sequencing analysis such as GRO-seq [7] would be more appropriated as transcriptomic assays reflect the steady-state levels of transcripts.”*

*And also, the following sentences at Materials & Methods:*

*“The diff wig output was used to extract nucleosome occupancy difference between Epimastigote and TCT. The dSSR bed file (depicting their genomic coordinates) was used, along with the pyBigWig library, to extract the occupancy difference values for each dSSR and to calculate their average value”.*

*And*

*“ A bed file containing all polycistron genomic coordinates and the normalized TPM wig files of the transcriptomic study [38] were used to obtain polycistronic TPM counts by using the annotatePeaks function from HOMER [74].*

*”*

Reviewer #3: (No Response)

Part III – Minor Issues: Editorial and Data Presentation Modifications

Please use this section for editorial suggestions as well as relatively minor modifications of existing data that would enhance clarity.

Reviewer #1: Corrections:

1) P11, L248-249: Sentence reads:

"These findings suggest an unpredict regulation maintained in T. cruzi chromatin reflecting phenotypic differences between replicative and nonreplicative forms."

It is unclear what the authors intend with this sentence and should be clarified.

*The fact that GO terms associated with “regulation of nuclear division”, “regulation of cell division”, “regulation of translation”, “chromatin remodeling”, “adhesion to a symbiont host cell”, and “antigenic variation” are enriched amongst the genes/polycistrons associated to dynamic dSSR is very interesting once suggest that phenotypic differences found in these life forms (cell cycle competence x cell cycle arrest; high translation rate x low translation rate; expression of virulence factors x no expression; adhesion to substrate x no adhesion) is (somehow) depicted at the gene locus associate to these phenomena. Once trypanosome gene regulation is held mainly at post transcriptional mechanisms, it seems an unpredicted finding. We have clarified the sentence as follow:*

*“Once gene expression is mainly regulated by posttranscriptional mechanisms, these findings suggest an unpredict regulation maintained in T. cruzi chromatin reflecting phenotypic differences between replicative and nonreplicative forms as it will be discussed later.”*

2) Figure 1E and S6 shows the percentage of dynamic and static nucleosomes at different genomic features gene classes. It is unclear from the text and figure legends if these graphs are representative of epimastigotes, TCTs, or an average of the two life forms.

*One of the DANPOS output includes the sum of all nucleosomes identified at both life forms. Thus, figure 1E and S6 were based on this dataset, which includes nucleosomes with the same genomic location and those that are different between life forms. We clarified this part adding the following sentence at Figure 1E and S6 legends:*

*“…(sum of nucleosomes from both life forms).”*

3) Figure 2D shows GO terms associated with dynamic nucleosomes. One GO term contains a typo, reading "regulation of mytotitiv cytokinesis", that should be corrected.

*Thanks, we have corrected.*

4) Figures 2D, 3A, and S8B show GO term enrichment. The y and x axis are labelled "semantic space" with scales. It is unclear what these labels and scales represent and should be either explained in the appropriate legends, or described more clearly in the figures to be easier to follow.

*REVIGO’s paper described that semantic space relies on semantic similarity measures [9]. According to the authors “Both the summarization and the visualization step draw on the concept of GO term semantic similarity, reviewed in [10]” In general, the more semantically similar GO terms the more closer they are plotted.*

*Thus, we added the following sentence on legends of Fig 2,3 and S8.*

*“The scatterplot shows a clusterization of GO terms (remaining after a redundancy reduction) in a two-dimensional semantic space, resulting in similar terms being plotted next to each other according to [9].  In general, more semantically similar GO terms are closer in the plot.”*

5) In the text, figure 3C is mentioned before 3A and 3B - authors should rearrange the figure panels so that data is presented in sequential order.

*We have rearranged figure 3 as suggested and corrected the legends accordingly.*

6) Figure 8A:

The legend for figure S8A reads: "...(filtered by those whose GO terms harbor f than 8 members)." This sentence should be clarified.

*We corrected by “(filtered by those whose GO terms harbor fewer than 8 members).”.*

It continues "..IDs are shown in the Supplemental Table." Table S5 should be mentioned here.

*Ok. We have indicated the table’s number (S5).*

The first panel of fig 8A reads "6980 static IDs", while the legend refers to "nondynamic IDs". Authors should use one of these terms consistently.

*We have changed nondynamic IDs for static IDs, which is more meaningful.*

The second panel of fig 8A reads "3499 dynamics ID", one presumes this should be "dynamic", not the plural form.

*We have corrected.*

7) Figure 4:

P14, L341 - 345: This sentence presumably refers to fig 4B, and should state it in the text.

*We thank the referee for this comment however the sentence refers to Figure 4A. We have stated it in the text.*

P15 L347 - 349 discusses the nucleosomal landscape around TSSs and TTSs in the polycistronic environment and refers to fig 4B. From the fig 4 legend, it appears that this sentence should refer to fig 4 C and D, not B.

*Yes, the referee is right, and we have corrected the text.*

8) Fig 5:

Panels A - G should be included in legend

*Thanks, we included.*

Panel 5H is not referred to in text and may be more appropriate in the supporting information section.In addition, adding an IGV snapshot showing the data presented in 5H over a larger genomic region (100 kb) and indicating the PTU directionality (as in fig 2A) will provide a great overview of the NPO data. This will benefit the paper as well as the reader.

*We have moved panel 5H top to the supporting information (new Figure S9) together with an IGV snapshot over a larger genomic region as suggested. We prefered to maintain the figure 5H bottom at the main figure as it confirms data from figure 5E and 5F, which show that tRNA and snoRNA have different abundance of nucleosomes in life forms. However, as suggested by the referee we also provided a similar IGV snapshot over a larger genomic region for figure 5H bottom. In addition, now we have mentioned Figure 5H in the text.*

9) On the y-axis of fig S9C, "cSSR_center-intergenic_center" is partly cut off in figure.

*We have fixed this problem.*

10) P17 L399 - 401 reads: "In addition, when normalized by total length in kbp, DGF-1 genes had more dynamic nucleosomes per kbp than all other gene classes (Fig S10B)."

Fig S10B is labelled '# nuv dynamic per ID' and does not make sense in the context of what is being described. If the authors instead refer to the second panel of S10A, this statement makes more sense. From the legend it seems that the description of B fits to the second panel of A.

If the above is correct, the panel currently labelled B is not described in the legend or mentioned in the text. It appears that this panel is referred to in L405 - 406. The authors should rectify this.

*We have corrected the legend accordingly (previous S10 figure is now S11). Indeed, legend for S11B described S11A (bottom). We have clarified it as well as described S11B on the legend as “****B.*** *Number of dynamic nucleosomes per ID at the same categories described above.” In addition, now Fig S11B is mentioned on the text.*

*“Here, we detected that this gene class is also more likely to have dynamic nucleosomes (Fig S11B).”*

11) P18 L423 reads: "...as shown in Fig 6D." This should be 6C.

*We have corrected.*

12) P18 L434 indicates the authors classified the transcripts into 5 groups namely: highest, high, medium, low, and differential. However, figure 7 and its legend (P28 L688 - 693) only refers to 4 groups - high, medium, low, and differential, not highest. Authors should clarify this.

*Only 4 groups were classified: high, medium, low, and differential; therefore, we have corrected the text.*

13) P26 L632 indicates that chromatin was digested with 1500U MNase. Authors should indicate whether this is in Sigma or Worthington units, as one unit Sigma MNase has the same activity as 85 Worthington units.

*We have used micrococcal nuclease from Thermo (Part No. 88216), whose unit is defined (according to Thermo website) as follow: 1 unit is defined as the amount of enzyme required to produce an increase in absorbance at 260nm of 0.001/min/mL at 25°C of highly polymerized DNA.*

*The information of which company the enzyme belongs was missing and now we have completed to avoid misunderstandings.*

14) P27 L644 - authors should state the read length of paired-end reads obtained from Illumnina sequencing.

*It was 2 x 75 bp. We have added this information at Materials & Methods.*

Reviewer #2: MNase-seq data is presented as IGV snapshots in three figures (Figs 1, 2 and 5), but in all cases it is impossible to fully evaluate these data. Most importantly, the authors need to provide values for the MNase-seq mapping in order that the extent of read depth can be seen, in order that we can understand how epimastigotes and trypomastigotes compare, and how large the differences (‘diff’) are. In addition, the other genomic features detailed, plus the meaning of dynamic and how his was assigned, needs explained to the reader; without this information, these figures are very hard to follow.

*IGV snapshots are representative examples of the findings. We have now added the scales for both read depth and diff at all IGV snapshots. In fact, the numbers were described on the figures however they were too small.*

*I’m afraid I haven’t understand what reviewer means by “the meaning of dynamic and how his was assigned, needs explained to the reader;” in the context of IGV snapshots. Does it refer to the “diff plot” at IGV or how Danpos calculate dynamics nucleosomes?*

*If reviewer refers to Diff plot:*

*“The diff wig file indicates the occupancy difference at each base pair between the epimastigote and TCT data”, which were previously stated at Fig 2 legend. Now, we have complemented this information with “The diff wig file indicates the occupancy difference at each base pair between the epimastigote and TCT data: red means more occupancy on epimastigote and blue means more occupancy on TCT”.*

*If reviewer refers to how dynamic nucleosomes were calculated by Danpos:*

*Dynamic nucleosomes were previously described at M&M : “In short, DANPOS2 calculates nucleosome occupancy in two samples and performs a differential test (Poisson by default) to detect signal variations at a given genomic location”. In addition, at Figure S4B a scheme of dynamic nucleosomes classes obtained from DANPOS was depicted.*

*According to DANPOS paper: “DANPOS is a statistical pipeline explicitly designed for the analysis of dynamic nucleosomes. Most of the previous analysis programs have been devoted to nucleosome calling from only one experimental condition. However, much less attention has been paid to the dynamic changes of nucleosomes between different conditions, such as disease vs. control“*

*We added the following sentence at Results part:*

*“This latter (**dynamic nucleosomes) is inferred by optimizing data normalization and statistical testing between two samples”*

Lines 423-430. I’m afraid that I am not convinced by the authors’ suggestion that ‘MASP, TS and RHS genes have a similar landscape with a clear NDR near the first ATG followed by a well-positioned nucleosome downstream and a fuzziness pattern’, [which differs from] ‘DGF-1 and GP63 [which] are more similar to each other, with an NDR just upstream of the first ATG, followed by an increase in nucleosome occupancy all along the gene body’. Visually, I cannot see this difference between the 2 gene groups, which all appear very similar; to verify this, the authors need to more rigorously compare the MNase-seq profiles between the gene families. In addition, why are mucins not discussed, since the nucleosome pattern for this gene family looks different to all the others.

*We have rearranged the figures to facilitate comparison. Additionally, we have mentioned mucin nucleosome landscape on the text, as follow “ Mucins have a nucleosome landscape that seems different from both groups”.*

Reviewer #3:

Page 8 - Line 84

What would be the meaning of: "most nucleosomes are poorly positioned."

*Some authors refer to “poorly positioned” nucleosomes those that are not positioned at the same genomic location in a cell population. The classification of fuzziness, which will be defined later on these manuscript (…“fuzziness (fuzz)” reflect the standard deviation of nucleosome position in different cells: a low standard deviation means less fuzziness and more well-positioned nucleosomes, while a high standard deviation means more fuzziness and a dispersed nucleosome pattern”), meets more clearly and with a mathematical criteria, the meaning of poorly positioning.*

*We agree with the referee that the sentence is confusing and therefore we changed “poorly positioned” by “most nucleosomes have a fuzzy pattern”.*

Page 9 – line 101: . . . that function as TSSs and TTSs (transcription termination sites) [11, 12]. missing shouldn't it be: "transcription initiation and transcription termination sites, respectively"?

*Ok, we have corrected.*

Page 9 – line 103: 5'end is a more precise term than 5'portion.

*Ok, we have corrected.*

Page 9 – line 105: "gene expression is remarkable," shouldn't it be something similar to "the control of gene expression which is remarkable."

*We are not just referring to the control of gene expression but also the mechanisms that are important for gene expression, such as polycistronic transcription, the coordination of trans-splicing and polyadenylation etc. Therefore, we would prefer to maintain the text as it was.*

Page 9 – line 110: Instead of "A second interesting biological feature," wouldn't it be better to use "Another interesting biological…"?

*Ok, we have changed the text.*

Page 17 – line 307: there is an incorrection "two DNA topoisomerases from classes II and II."

*Only two DNA topoisomerases were found. As we do not discuss differences among classes, we removed the sentence “from classes II and II” to avoid misunderstandings.*

Page 17 – paragraph starting on line 306: A general comment regards the citation of genes by their TriTryp DB identity. It turns out that reading a text with these TriTryp identities is not agreeable and nor informative. Also, in the same paragraph: what is a "generic ribosomal protein"?

*We agree that reading IDs is not agreeable, however I see no other way to distinguish between genes that have the same description but different IDs (and therefore, different genomic coordinates). The “generic ribosomal protein” was originally the description stated at TriTypDB, however, a search at currently version indicates that TcCLB.510879.20 as “ribosomal protein S19, putative” and TcCLB.511545.40- as “ribosomal protein L27, putative”. TritrypDB classifies ribosomal proteins into, at least, 4 large groups: ribosomal proteins and 40S, 50S or 60S ribosomal proteins. I could not find an explanation why some proteins are classified as only ribosomal proteins while others are named by the rRNA that they are associated. We have removed the term “generic” and mentioned their current description name accordingly.*

Page 26 – lines 520-524: The sentence starting on "In silico analysis of RNA Pol II component expression from public . . ." is confusing. More importantly, if the authors are not inclined to present their data, they should eliminate this sentence.

*Due to high amount of data showed in this paper, we choose not to plot a graph showing the expression of RNA Pol II subunits. Thus, as we are not going to show the data, we have removed this sentence as suggested.*

PLOS authors have the option to publish the peer review history of their article (what does this mean?). If published, this will include your full peer review and any attached files.

If you choose “no”, your identity will remain anonymous but your review may still be made public.

Do you want your identity to be public for this peer review? For information about this choice, including consent withdrawal, please see our Privacy Policy.

Reviewer #1: Yes: Hugh -G Patterton

Reviewer #2: No

Reviewer #3: No

Figure Files:

While revising your submission, please upload your figure files to the Preflight Analysis and Conversion Engine (PACE) digital diagnostic tool, https://pacev2.apexcovantage.com. PACE helps ensure that figures meet PLOS requirements. To use PACE, you must first register as a user. Then, login and navigate to the UPLOAD tab, where you will find detailed instructions on how to use the tool. If you encounter any issues or have any questions when using PACE, please email us at [figures@plos.org](mailto:figures@plos.org).

*This new version contains the PACE corrected figures files.*

Data Requirements:

Please note that, as a condition of publication, PLOS' data policy requires that you make available all data used to draw the conclusions outlined in your manuscript. Data must be deposited in an appropriate repository, included within the body of the manuscript, or uploaded as supporting information. This includes all numerical values that were used to generate graphs, histograms etc.. For an example see here: [http://www.plosbiology.org/article/info%3Adoi%2F10.1371%2Fjournal.pbio.1001908#s5](http://www.plosbiology.org/article/info%3Adoi%2F10.1371%2Fjournal.pbio.1001908#s5).

*The datasets generated and/or analyzed during the current study are available in the NCBI BioProject repository, under the accession code PRJNA665060 and BioSample codes SAMN16241276, SAMN16241277, SAMN16241279, SAMN16241280, SAMN16241281 and SAMN16241282. In addition, we provided an excel file containing the numerical values used to generate graphs. Of note, this article contains 5 supp Tables, which contains data that were used to build the graphs and conclusions of this manuscript. Besides, the great majority of our bar graphs show all absolute (and/or percentage) values. The GO analysis (values, scores, etc) from Fig 3A, C and S8 are described at Table S5. The GO analysis (values, scores, etc) from Fig 2D are described at Table S4, while polycistronic information is showed at Table S3. Table S2 contain data that were used to build the graphs from Fig1B-E, 2B-C and 6A-B.*

Reproducibility:

To enhance the reproducibility of your results, PLOS recommends that you deposit laboratory protocols in protocols.io, where a protocol can be assigned its own identifier (DOI) such that it can be cited independently in the future. For instructions see http://journals.plos.org/plospathogens/s/submission-guidelines#loc-materials-and-methods

1.	Pays, E., et al., *Abrupt RNA changes precede the first cell division during the differentiation of Trypanosoma brucei bloodstream forms into procyclic forms in vitro.* Mol Biochem Parasitol, 1993. **61**(1): p. 107-14.

2.	Christiano, R., et al., *The proteome and transcriptome of the infectious metacyclic form of Trypanosoma brucei define quiescent cells primed for mammalian invasion.* Mol Microbiol, 2017. **106**(1): p. 74-92.

3.	Liu, B., K. Kamanyi Marucha, and C. Clayton, *The zinc finger proteins ZC3H20 and ZC3H21 stabilise mRNAs encoding membrane proteins and mitochondrial proteins in insect-form Trypanosoma brucei.* Mol Microbiol, 2020. **113**(2): p. 430-451.

4.	Siegel, T.N., et al., *Four histone variants mark the boundaries of polycistronic transcription units in Trypanosoma brucei.* Genes Dev, 2009. **23**(9): p. 1063-76.

5.	Wedel, C., et al., *GT-rich promoters can drive RNA pol II transcription and deposition of H2A.Z in African trypanosomes.* Embo j, 2017. **36**(17): p. 2581-2594.

6.	Martínez-Calvillo, S., et al., *Transcription initiation and termination on Leishmania major chromosome 3.* Eukaryot Cell, 2004. **3**(2): p. 506-17.

7.	Gardini, A., *Global Run-On Sequencing (GRO-Seq).* Methods Mol Biol, 2017. **1468**: p. 111-20.

8.	Li, Y., et al., *Transcriptome Remodeling in Trypanosoma cruzi and Human Cells during Intracellular Infection.* PLoS Pathog, 2016. **12**(4): p. e1005511.

9.	Supek, F., et al., *REVIGO summarizes and visualizes long lists of gene ontology terms.* PLoS One, 2011. **6**(7): p. e21800.

10.	Pesquita, C., et al., *Semantic similarity in biomedical ontologies.* PLoS Comput Biol, 2009. **5**(7): p. e1000443.
