# Supplementary material for: Nucleosome landscape reflects phenotypic differences in Trypanosoma cruzi life forms
Source: PLoS Pathog. 2021 Jan 26;17(1):e1009272. doi: 10.1371/journal.ppat.1009272 (PMC7864430; doi:10.1371/journal.ppat.1009272)
Supplement: Attachment — Submitted filename: AnswersFinal2812.doc [file ppat.1009272.s019.doc]

**São Paulo, 12/28/2020**

**Dear editor,**

**Dr. Christine Clayton**

We thank the editors and the referees for suggestions on our manuscript. I corrected the 5 points suggested below:

1. "Once gene expression is mainly regulated by posttranscriptional mechanisms" should be "Since gene expression...

   2. The phrase "an unpredict regulation" doesn't make sense. Do you mean "unexpected regulation"? (Actually I'm not even sure "unpredict" exists, but if it does the meaning isn't appropriate.)

   3. "Mucins show a nucleosome landscape that look different" should be "looks different".

   4. "the steady-state levels of transcripts is the result of the balance among transcription expression, expression, RNA degradation and stabilization" should be
   "are the combined result of transcription, RNA processing, and mRNA degradation". (Stablization affects degradation so isn't a separate contributor; and processing has to be mentioned.)

   5. Line 504: delete: "as soon as they were identified in T. cruzi". Alternatively you could say: "since such sites have not yet been mapped in T. cruzi".

            Please do not hesitate to contact us if you need any further information.

            Sincerely,

Julia Cunha, PhD

Av. Vital Brasil, 1500. CEP: 05338-000
